# Development of a text-messaging intervention to improve treatment adherence and post-treatment review of children with uncomplicated malaria in western Kenya

**DOI:** 10.1186/s12936-015-0825-x

**Published:** 2015-08-19

**Authors:** Sophie Githinji, Caroline Jones, Josephine Malinga, Robert W Snow, Ambrose Talisuna, Dejan Zurovac

**Affiliations:** Department of Public Health Research, KEMRI-Wellcome Trust-University of Oxford Research Programme, Nairobi, Kenya; Centre for Tropical Medicine & Global Health, Nuffield Department of Clinical Medicine, University of Oxford, Oxford, UK; Center for Global Health and Development, Boston University School of Public Health, Boston, MA USA

**Keywords:** Text-messaging, mHealth, Adherence to ACT, Intervention development

## Abstract

**Background:**

Patients’ low adherence to artemisinin-based combination therapy has been reported in areas of Kenya bordering the Lake Victoria region, where the burden of malaria remains high. A randomized controlled trial is underway to determine the efficacy of short message service (SMS) text reminders on adherence to artemether-lumefantrine and post-treatment review of children under the age of five. This paper reports on the iterative process of intervention and delivery system development.

**Methods:**

An intervention development workshop involving the research team and other stakeholders was held to determine the content of the text messages. Three focus group discussions were conducted to test caregivers’ understanding of the messages developed during the workshop. The tested messages were refined and incorporated into an automated SMS distribution system and piloted with 20 caregivers drawn from facilities neighbouring the study sites. The automated SMS distribution system was repeatedly refined following the pilot and implemented at the start of the trial.

**Results:**

The content of SMS messages underwent major revisions following the focus group discussions. Technical terms and abbreviations were replaced with simplified general terms. Message sign-off was modified to reflect the name of health facility, removing references to health workers. Day 3 post-treatment review visit reminder was modified to state the purpose of the visit while wording ‘day 28’ was added to the last post-treatment review visit reminder to help the caregiver recall the appointment date. The unscheduled visit prompt was modified to reflect flexibility and practicality of taking the child back to the facility if unwell. Reception of SMS reminders during the pilot was low with only 169/240 (70%) of scheduled messages delivered to the caregivers. The automated distribution system underwent major refinement and repeated testing following the pilot until effective delivery of all scheduled messages was achieved and sustained over a period of 3 months.

**Conclusions:**

Text message interventions should be carefully developed, tested and refined before implementation to ensure they are written in the most appropriate way for their target population. SMS distribution systems should be rigorously tested to ensure efficient delivery of the messages before they are deployed.

## Background

The World Health Organization (WHO) has recommended adoption of artemisinin combination therapy (ACT) for the treatment of uncomplicated falciparum malaria in 2006. Consequently, increasing quantities of effective ACT are available worldwide, with Africa accounting for 95% of treatments distributed in 2013 [1]. Artemether-lumefantrine (AL) is a fixed-dose combination ACT that is most widely procured and distributed worldwide. However, low rates of patients’ adherence to ACT regimens have continuously been reported across different settings [2–7]. Failure to complete therapeutic doses increases the risk of clinical failure and could contribute to the development and spread of drug resistance, threatening the sustainability of current anti-malarial efforts [8–10]. Strategies to improve patient adherence to the full therapeutic course of malaria treatment are urgently required [9, 11]. Interventions already applied include the use of packaging aids, visual media, verbal information, convenient regimen with either once daily or short treatment duration, community education, and medication supervision [12]. With the rapid growth of mobile phone subscriptions, reaching 6.9 billion in 2014, and 90% penetration in developing countries [13], use of short message service (SMS) text messaging is presenting a new type of intervention to facilitate patient communication and potentially improve adherence to treatment dosing schedules [14].

Short message service reminders have been found to improve adherence to antiretroviral therapy (ART) in studies conducted in different settings [15–18]. However, few studies have evaluated the effects of SMS reminders on adherence to ACT [19, 20]. Randomized controlled studies are required to build the evidence base of using patient-centred SMS reminders on adherence to ACT. In 2014, a multicentre, randomized, controlled trial (SMS-RES-MAL) was established to determine the efficacy of using SMS text message reminders to improve patient adherence to AL and post-treatment review for children under 5 years old [21]. The trial is taking place in Siaya County, Kenya, an area with reported low adherence to ACT prescription [22]. Despite the growing body of literature documenting the outcomes of SMS-based interventions, few studies have reported detailed descriptions of how those interventions were designed, developed and implemented [23]. This paper reports on formative research conducted in the first phase of the trial. The primary objective of the formative research was to develop, pilot and refine an automated text messaging intervention to promote adherence to AL.

## Methods

### Description of the study area

The SMS-RES-MAL trial was established at four public health facilities in Siaya County. Malaria contributes to 54% of morbidity in Siaya County and is transmitted throughout the year [24]. Prevalence rates for *Plasmodium falciparum* among children aged 2–10 years (*Pf*PR_2-10_) are predicted to be 50% across Siaya County [25]. A feasibility study conducted in 2013 showed high access to mobile phones within the households across the catchment areas of the study facilities (93.0%), high use of SMS functions to receive messages (93.6%) and almost universal (99.7%) willingness to receive text messages on child health [26].

### Study design

The study is conducted in three phases. Phase 1, completed in 2014 and reported in this paper, involved developing and piloting the text messaging intervention and automated distribution system. Phase 2, currently on-going, is the randomized control trial involving 2,000 children to assess the efficacy of the SMS intervention [21]. Phase 3 will be conducted at the end of the trial to describe the factors contributing to the success or failure of the intervention.

### Development of the intervention

**Fig. 1:**
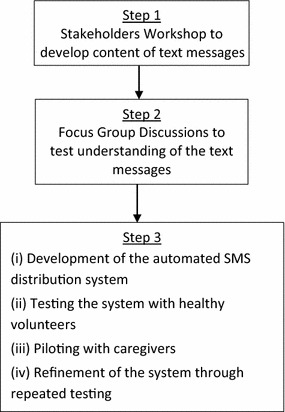
Intervention development process.

The recommended first-line treatment for uncomplicated malaria in Kenya is AL. Recommended dosing schedule of AL is twice daily every 12 h over 3 days, with an exception of the second dose which should be administered after 8 h. The intervention developed for this study was a one-way, automated distribution of SMS reminder messages on dose administration and post-treatment review visits. These reminders were sent to caregivers of children under the age of 5 years treated for confirmed uncomplicated malaria. Dose administration reminder messages were sent to coincide with the recommended dosing schedule. Post-treatment review reminders were sent on the scheduled review days: day 3 and day 28 and weekly for unscheduled visits, which prompted caregivers to take the child back to the health facility any time if unwell. This mobile phone-based intervention service was developed through a three-step process summarized in Fig. 1 and described as follows:

*Step 1: Stakeholders workshop* In September 2013, prior to the establishment of the trial, a two-day stakeholders’ workshop was held in Siaya County to develop the content of the text messages and determine their number, timing, language, and frequency. The workshop brought together community health workers, clinicians, district health management teams, mobile phone system developers and the research team (Table 1). The research team introduced the study, highlighting its purpose, objectives, procedures, and expected outcomes. Over the 2 days, the stakeholders held work sessions to develop the content of SMS messages covering four categories; (1) dose reminders; (2) day 3 post-treatment review; (3) unscheduled visit prompts; and, (4) day 28 review reminders. After each work session a plenary session was held to deliberate and reach a consensus on which messages to adopt for pre-testing. Ten messages were selected for pre-testing: two messages each for dose reminders, unscheduled visit prompts and day 28 review. There was no consensus on which messages to pre-test for day 3 post-treatment review, hence all four messages developed were taken for pre-test. All messages were developed in Dholuo, Kiswahili and English, the three languages commonly spoken in the study area. In addition, participants agreed upon appropriate times and frequency of sending the SMS reminder messages based on routine daily activities and work schedules of the study community (Table 2).

**Table 1:** Stakeholders involved in the intervention development workshop

Stakeholders’ designation	Number of participants
Mobile phone programming company	1
Clinicians	4
Community health workers	4
Research team	4
District health management teams	10
Total	23

**Table 2:** Scheduling of SMS reminders for the SMS-RES-MAL trial

Message reminder	Timing	Day of sending
AL dose 2^a^	8 h after first dose	Day 0
AL dose 3	8.00 am	Day 1
AL dose 4	8.00 pm	Day 1
AL dose 5	8.00 am	Day 2
AL dose 6	8.00 pm	Day 2
Day 3 post-treatment review visit	8.30 pm	Day 2
	8.00 am	Day 3
Unscheduled visit prompts	8.00 am	Day 7
	8.00 am	Day 14
	8.00 am	Day 21
Day 28 post-treatment review	6.30 pm	Day 27
	8.00 am	Day 28

^a^AL dose one is given at the health facility, directly observed by the health workers as per national guidelines.

*Step 2: Testing understanding of SMS reminder messages* Focus group discussions (FGDs) with caregivers were held to test understanding of the text messages developed during the stakeholders’ workshop. This method was the preferred approach as group discussions not only allow participants to express their individual understandings and interpretations of the messages, but also encourage discussion and the establishment of a consensus around the most appropriate content. Participants of the FGDs were recruited from among caregivers of children under 5 years treated for malaria at the four trial health facilities. The study team consecutively recruited the first seven consenting caregivers at each trial facility to participate in the FGDs in the language of their choice. Three FGDs were held, one each in Dholuo, Kiswahili and English (Table 3). Only women caregivers brought children to the facilities during the days of recruitment. Twenty-seven women aged between 18 and 45 years participated in the FGDs, each lasting between 45 and 60 min. The facilitating team consisted of a member of the research team (SG), a note taker and two community health workers (CHWs). The SMS messages were discussed under the four categories developed by the stakeholders. Participants were presented with one printed SMS message at a time and discussed: what they understood from the messages, any unclear or ambiguous terms and potential alternative wording to replace such terms. Before moving from one category to the next, participants deliberated to a consensus on the message that best communicated the intended information. All discussions were audio recorded and transcribed verbatim in Word. Kiswahili and Dholuo transcripts were translated into English and checked against their original language. A member of the research team (SG) checked, verified and coded the transcripts in NVIVO based on the four message categories. Analysis was restricted to key quotes that showed how the original messages were interpreted and transformed by the participants.

**Table 3:** Characteristics of participants of focus group discussions

Group	Number of participants	Age range	Mean age
English	10	23–32	27.1
Kiswahili	8	18–40	24.6
Dholuo	9	21–45	28.6
Overall	27	18–45	26.8

*Step 3: Development, testing and piloting of the automated SMS distribution system* An automated SMS distribution system for sending the reminder messages was developed by *Medic Mobile,* a technology company that builds mobile and web tools for use in health [27]. The system uses mobile phone technology to collect, aggregate and display health information. It is composed of (1) an SMS data collection tool which uses an in-built SMS gateway to receive and send structured SMS messages that are then stored in a local database; and, (2) an analytics tool that enables visualization and analysis of data sent via SMS to track operational progress of the trial. The SMS messages agreed upon during the FGDs were incorporated into the system together with unique patient randomization numbers mapped to the intervention and control arms of the study. Each study health facility was registered into the automated SMS distribution system together with its specific mobile phone number to register patients. Only registrations from the registered mobile phone numbers are accepted into the system. A study nurse registers a patient caregiver into the system by sending a structured text message to the designated gateway telephone number with details of patient’s unique randomization number, caregiver’s name, their mobile phone number and preferred language option. Upon a successful registration, the system creates a schedule of reminder messages to be sent as per the established timing (Table 2) for random numbers mapped to the intervention arm but does not create a schedule for the numbers mapped to the control arm. The system then sends a registration confirmation text message to the study nurse and the patient caregiver irrespective of their study arm allocation.

To test the automated SMS distribution system, 40 healthy volunteers were registered and followed up for 28 days to check if each scheduled message was delivered to their mobile phone as per the specified timing. The volunteers were recruited among colleagues of the study team based in Nairobi and Kilifi County and stakeholders living in the study area. The distribution system was then piloted with 20 consenting caregivers of children under five treated for uncomplicated malaria at four facilities neighbouring the study sites. The study team recruited the first five consenting and eligible caregivers at each site to participate in the pilot. The caregivers were registered into the SMS distribution system and visited at their homes on day 3 and day 28 to check whether they received the messages. The first visit checked the dose reminders and day 3 post-treatment review reminders while the second visit checked weekly unscheduled visit prompts and day 28 post-treatment review reminders. During each visit, the research team confirmed whether each scheduled message was delivered to the caregiver’s phone and recorded the date and time of delivery. If a scheduled message was not delivered, the research team probed the caregiver on whether they had deleted the message, if their mobile phone was on throughout and if there was cell phone network signal within their homestead. Caregivers were also asked if they read the messages and to explain briefly what they understood from each message.

### Ethical approval

The study was approved by the Kenya Medical Research Institute Ethical Review Committee; reference number SSC 2554. Written informed consent was obtained from all participants of the focus group discussions and the pilot.

## Results

### Testing understanding of the messages

*Dose reminders* Only dose-two reminders were discussed since all dose reminders had the same content, except for the dose number. Two messages were discussed in this category:

Message 1: Hello Maorine Aoko Ojwang, have you remembered to give your child the 2nd dose of AL? If not, please do so. Thank you, Doctor Bondo District hospital.
Message 2: Hello Maorine Aoko Ojwang, please remember to give your child the 2nd dose of AL. Thank you, Doctor Bondo District hospital.

The abbreviation “AL” in the two messages was not familiar to most caregivers.

“My worry is, not all of us know about the drugs so this AL, let’s say you have sent me this message and I don’t know what AL is, I will be confused.” [FGD, English].

Participants were concerned that most caregivers would not know the name of the drug they had been given.

“I still refuse this coartem, [name suggested to replace AL] for one reason, there are some ‘shoshos’ [grandmothers] at home but if you send the coartem, they will not read that coartem, but if you send anti malaria, the shosho will remember yes, my granddaughter sick with malaria so I think this is the same drug they are referring to.” [FGD, English].

Participants agreed to use a more general term ‘malaria medicine’ as opposed to specific drug names. Some participants also expressed concerns that message two could lead to overdose if the caregiver received the message after they had already given the required dose.

“Yeah, with the first one, [message one] the doctor first confirmed with me if I have already given [the dose] or not, then he urged me to do so if I had not done it. But this one [the second message] is reminding me to remember. Suppose I had given the baby the dose the medicine… So I don’t know whether to give so, I might give the second dose when I have already given… I prefer the first one because the person has asked the person if the person has already given the drug and if not please do so.” [FGD, English].

In addition, message one was perceived to be more forceful as it asks a question “have you…?” and directs an action “If not, please do so” if the caregiver had forgotten to dose their child.

“Me, I see the second one doesn’t the person a lot, when you read it, it does not have that seriousness, like me I have got it and took it just like that, because it is requesting me, but when you read the first one, Have you? It is like asking you a question, it is a question to me, it is attacking me direct.” [FGD, Kiswahili].

Given the preference for message one and concerns raised about possibility of confusing the caregiver if a specified dose had already been given before receiving the message, message one was adopted with an amendment replacing “AL” with “malaria medicine” as suggested by the groups (Table 4).

**Table 4:** SMS messages adopted following the focus group discussions

Message category	Message content
AL dose reminders	Hello [name of care giver], have you remembered to give your child the [dose number] dose of malaria medicine? If not, please do so. Thank you, [Name of health facility]
Day 3 post-treatment review reminder	
Evening	Hello [name of care giver], please remember to bring the child back to hospital tomorrow to confirm clearance of malaria parasites. Thank you, [Name of health facility]
Morning	Hello [name of care giver], please remember to bring the child back to hospital today to confirm clearance of malaria parasites. Thank you, [Name of health facility]
Unscheduled visit prompt	Hello [name of care giver] I hope the child is doing well. If not, please bring them back to the hospital as soon as possible. Thank you, [Name of health facility].
Day 28 post-treatment review reminder	Hello [name of care giver], please bring your child back to the hospital tomorrow for day 28 review as advised by the doctor. Thank you, [Name of health facility]
	Hello [name of care giver], please bring your child back to the hospital today for day 28 review as advised by the doctor. Thank you, [Name of health facility]

*Day three post-treatment review reminders* Four messages were discussed:

Message 1: Hello Mary Akinyi Bukhula, please remember to bring your child back to the hospital tomorrow for review. This is important to check if the child is free from malaria. Thank you, Doctor Bondo District hospital.
Messages 2: Hello Mama Junior, thank you for finishing malaria treatment for Junior, please remember to bring back the child for review tomorrow as we had agreed. Thank you, Got Agulu SDH.
Message 3: Hello Michael Ojwang, thank you for giving AL dose to your child as advised by the doctor, please bring your child back to the hospital tomorrow to confirm if the malaria parasites have cleared. Sister Ndori Health Centre.
Message 4: Hello Mary, please bring your child to Ndori Health Centre for review tomorrow to check if the child is free from malaria. Thank you, Sister Ndori.

Participants raised concerns about the assumptions made in messages two and three.

[Referring to message two] “The doctor is assuming that you had finished the dose that you were given… but there is a problem, why? Most mothers we do assume or negligence or I don’t know how to call it but once you see the child now can play, can laugh, even if you get the message you will not go you assume that the child is now okay.” [FGD, English].

There were also concerns about how messages three and four were signed off. Many of the participants preferred to use the health facility name instead of health care provider citing poor relationship between caregivers and individual health care providers.

[Message three] “Some health workers are always very arrogant until maybe they make the mother feel very bad to go back to the facility for treatment because they know sister so and so is there she will treat me the same way she treated me the other day, so this always, there is stigma for most mothers they don’t go back to the facility because of that.” [FGD, English].

[Message four] “Write full name, one can go to the wrong hospital…Hospital is more important than saying sister, sister Ndori, say Ndori Hospital.” [FGD, Kiswahili].

Participants also expressed a preference for messages that addressed the caregiver by their specific name rather than in general terms, such as *‘*mama’.

“The first [message one] is better, it indicates the name Akinyi. Mama Junior could be many, if you ask for mama Junior, it is not specific.” [FGD, Dholuo].

There was general consensus that stating the purpose of the visit added weight to the message and the mention of “malaria parasites” in message three was felt to reinforce the need to take the child for review:

[Referring to message three] “He [the doctor] wants to confirm if malaria is finished in the child, that’s why he wants you to take the child back, he wants to follow up…they have told you the importance of taking the child back…the other one [referring to message two] was open, it was just telling us to take the child back but maybe others will wonder why take the child back, I have finished the dose, but this one is telling us the why…we are going to confirm that malaria parasites are finished.” [FGD, English].

Taking into consideration all issues raised by the groups, the final message combined the first part of message one: (Hello Mary Akinyi Bukhula, please remember to bring your child back to the hospital tomorrow) and the second part of message three: (to confirm if the malaria parasites have cleared). Since the message was to be sent in the evening of day 2 and morning of day 3, the word “tomorrow” was replaced with “today” in the morning message. Following concerns raised regarding signing off the messages with “sister”, it was decided to sign off all messages with the name of the health facility only (Table 4).

*Unscheduled visit prompts*

Message 1: Hello Mary Akinyi Bukhula, I hope the child is well. If not, please bring the child back to the hospital for review. Thank you, Doctor Bondo District hospital.
Message 2: Hello Mama Junior, I hope your child is doing well. If not, please bring the child the back to the hospital immediately. Thank you, sister Got Agulu SDH.

The key concern about these messages was the use of the term “immediately” in message two. Many of the participants felt this was demanding and unrealistic, citing lack of transport especially at night and the fact that health facilities are not open 24 h, 7 days a week.

“Comment, comment here is immediately, by immediately, let’s say the child was sick last night, when you say immediately, will you attend to the client even at night?” [FGD, English].

However some caregivers thought the use of the word “immediately” conveyed a sense of urgency and added weight to the message;

“Okay to me the way I understand it, this term immediately, it shows that emergency, you when I as a mother, I will read this message and read immediately. …to me I see there is some emergency on it, so I will rush to the hospital … I think this word immediately will help a lot because if the mother if the child is still sick, the mother will say ah, I will go tomorrow. The child is becoming weak, let me give this child panadol, then I finish my work, then I will go tomorrow again. The child is still weak, now this immediately will help a lot”.

Since the two messages were very similar in content and the only contention was the practicality of bringing the child to the facility “immediately”, the term was replaced with “as soon as possible”.

*Day 28 post-treatment review reminder*

Message 1: Hello Mary Akinyi Bukhula, please remember to bring your child again tomorrow to hospital for reassessment. Thank you, Doctor Bondo District hospital.
Message 2: Hello Mama Junior, please remember to bring your child back to hospital tomorrow as we agreed, Sister Got Agulu SDH.

The term “reassessment” in message one was not clear to the participants and they suggested the addition of the term “malaria” to remind the caregiver what was being reassessed.

“To me, I see that reassessment is not so open…me I see is like you should stress that reassessment at least to give it a little weight … “Me I see this one is still hanging…you could have added something.” [FGD, Kiswahili].

In addition, several participants suggested that a mention of “day 28” should be added to the message to help the caregiver recall about the advice given to return the child on day 28. Message two was felt to contain connotations in the expression “as we agreed” that could negatively affect the caregiver.

“My husband will wonder if the doctor says ‘as we had agreed’. It can be misinterpreted” [FGD, Dholuo].

Consequently the expression “as we agreed” was rephrased to “as advised by the doctor” and a reference to “day 28” was added to the message (Table 4).

Following the FGDs, ten messages were adopted from each language: five dose reminders each specifying the dose number; two (morning and evening) day 3 post-treatment review visit reminders; one unscheduled visit prompt; and, two (morning and evening) day 28 review reminders. To check consistency across the three languages, a native speaker of Dholuo and a Kiswahili language expert back-translated the messages into English. A member of the research team (SG) compared back-translated messages with the ones developed in English and ascertained that the content and meaning was identical across the three languages.

### Piloting and refinement of the automated distribution system

Only 169 out of 240 (70%) scheduled messages were delivered to the piloted caregivers. None of the caregivers in the pilot received all the 12 scheduled messages. Fourteen of the 20 caregivers piloted received reminders for dose 2, 3 and 5; 18 received dose four reminder and 19 received reminder for dose 6. While all piloted caregivers received day 3 post-treatment review reminders, only five of them received all the scheduled dose reminders. The weekly unscheduled visit prompts for day 7, 14 and 21 were received by 12, 14 and 15 caregivers, respectively. Day 28 evening and morning reminders were received by 19 and 17 caregivers, respectively. Only eight caregivers received all unscheduled visit prompts and day 28 review visit reminders. Eighteen of the 20 caregivers piloted read the messages by themselves and correctly interpreted their meaning. Two caregivers had the messages read and interpreted for them correctly by another member of their household.

A meeting was held between the research team and the system programmers to discuss the pilot outcome. Poor Internet connectivity affecting transmission of scheduled messages from the web-based system to the in-built gateway and bugs within the system were established as the main reasons leading to the low reception of the messages during the pilot. Connectivity was resolved by relocating the gateway to a server room with reliable Internet. Bugs within the system were identified by continuously testing, troubleshooting and monitoring of the system. Over 100 healthy volunteers were repeatedly registered into the system over a period of 3 months and followed up to ascertain whether they received each message as scheduled. The research team notified the system developers of any messages that were delayed or not sent. The developers checked the system to troubleshoot and fix the errors. This process was continued until the system consistently sent all the messages to each registered volunteer as per the established schedule. Finally, to improve on system monitoring, an application was added to the system to send SMS notifications to the research team whenever there was a failure in the system.

## Discussion

Studies investigating SMS-based interventions in low-income settings have recommended more extensive research to better understand the most effective content of text messages to increase the benefits derived from mobile health applications [19, 28]. This paper reports on formative research conducted to inform the development of an automated text messaging intervention to improve adherence to AL treatment and to post-treatment review visits among caregivers of children treated for uncomplicated malaria. The findings of the study show the importance of obtaining feedback about the content of text message interventions and of rigorously testing their distribution systems before they are implemented. Poor internet connectivity and bugs within the system affected delivery of the text messages during the pilot. This led to repeated testing of the distribution system by both the research team and the system developers until it achieved efficient delivery of all scheduled messages. A study in Mozambique reported a similar experience and recommended close collaboration between the research teams and technology developers in defining the requirements, testing and refining of text message distribution systems before they are implemented [29].

The text messages developed by different stakeholders in this study were substantially revised by caregivers participating in focus group discussions conducted to test understanding of the messages. The discussions revealed ambiguities, assumptions and unfamiliar terms used in the messages. Terms like ‘AL’ and ‘reassessment’ were replaced with words that were more general and easy to understand. Similarly, a message that required a caregiver to take the child to the hospital immediately if unwell was revised to take into consideration circumstances like lack of transport if a child fell sick at night. Messages that could have multiple interpretations were identified and revised. Participants of FGDs preferred personalized SMS messages addressing the caregiver by their specific name. This informed the decision to programme the automated distribution system to send each message with a salutation addressing the caregiver by their name and language of choice. Using their specific names made the caregivers feel that the message was addressing them personally, even though the messages were not tailored to their individual needs. These findings are cognizant with reports that text message interventions are more likely to work when the message is personally tailored and when the frequency, wording and content are highly relevant [30]. A systematic review on efficacy of mobile phone interventions to improve medication adherence reported similar results where studies using SMS messages with personalized or tailored content (achieved thorough using the patient’s name, clinical condition, participants chosen content, and delivering the messages in different languages), improved medication adherence, as opposed to those with basic repetitious content [31]. Other studies evaluating patient education interventions have reported improvement in adherence to anti-malarial treatments when simple, easy-to-understand language was used to explain to patients how to take drugs [32, 33].

Caregivers preferred post-treatment reminder messages that explained the purpose of the follow-up visits to those that simply reminded them to take the child back to the health facility. The caregivers insisted on clear and convincing explanations to attend post-treatment reviews especially when the child was not sick. The effect of the text message reminders on post-treatment review attendance will be evaluated at the end of the trial. There is low evidence that mobile phone text message reminders improve attendance to health care appointments and more high quality research has been recommended to build this evidence especially in low income settings [34].

Participants in this study expressed reservations regarding signing off the messages with words that identified the message with particular health workers and instead preferred using health facility names. A review of behaviour change interventions using mobile phone text messages underscored the importance of understanding the nature of patient-healthcare provider interaction in designing SMS-based interventions [35]. Text messaging in health interventions is a form of communication between the patients and their healthcare providers, seeking to reinforce messages passed on during their face-to-face encounters. These messages should therefore be carefully formulated to enable effective communication that will lead the recipients towards the achieving the desired outcome. While a lot of emphasis is put on framing health messages in other behaviour change interventions [36], SMS text messaging is a relatively new type of intervention and it is not yet clear how best to formulate the messages to achieve the desired outcome [15, 17, 19]. Studies using SMS text messages should therefore incorporate methods of ensuring that the text messages are written in the most appropriate way for their target population [37]

## Conclusions

Text message interventions need to be carefully developed and tested to ensure they are written in the most appropriate way for their target population. Similarly, those wishing to use SMS distribution systems in health interventions need to work together with system developers to define the requirements, test functionality, refine and retest the systems before they are deployed.

## References

[1] WHO (2014). World malaria report: 2014.

[2] Ngasala BE, Malmberg M, Carlsson AM, Ferreira PE, Petzold MG, Blessborn D (2011). Effectiveness of artemether-lumefantrine provided by community health workers in under-five children with uncomplicated malaria in rural Tanzania: an open label prospective study. Malar J.

[3] Lemma H, Lofgren C, San Sebastian M (2011). Adherence to a six-dose regimen of artemether-lumefantrine among uncomplicated *Plasmodium falciparum* patients in the Tigray Region, Ethiopia. Malar J.

[4] Mace KE, Mwandama D, Jafali J, Luka M, Filler SJ, Sande J (2011). Adherence to treatment with artemether-lumefantrine for uncomplicated malaria in rural Malawi. Clin Infect Dis.

[5] Lawford H, Zurovac D, O’Reilly L, Hoibak S, Cowley A, Munga S (2011). Adherence to prescribed artemisinin-based combination therapy in Garissa and Bunyala districts, Kenya. Malar J.

[6] Minzi O, Maige S, Sasi P, Ngasala B (2014). Adherence to artemether-lumefantrine drug combination: a rural community experience six years after change of malaria treatment policy in Tanzania. Malar J.

[7] Depoortere E, Salvador ET, Stivanello E, Bisoffi Z, Guthmann JP (2004). Adherence to a combination of artemether and lumefantrine (Coartem) in Kajo Keji, southern Sudan. Ann Trop Med Parasitol.

[8] White NJ, Pongtavornpinyo W, Maude RJ, Saralamba S, Aguas R, Stepniewska K (2009). Hyperparasitaemia and low dosing are an important source of anti-malarial drug resistance. Malar J.

[9] Bruxvoort K, Goodman C, Kachur SP, Schellenberg D (2014). How patients take malaria treatment: a systematic review of the literature on adherence to antimalarial drugs. PLoS One.

[10] Banek K, Lalani M, Staedke SG, Chandramohan D (2014). Adherence to artemisinin-based combination therapy for the treatment of malaria: a systematic review of the evidence. Malar J.

[11] Brown MT, Bussell JK (2011). Medication adherence: WHO cares?. Mayo Clin Proc.

[12] Fuangchan A, Dhippayom T, Kongkaew C (2014). Intervention to promote patients’ adherence to antimalarial medication: a systematic review. Am J Trop Med Hyg.

[13] International Telecommunication Union (2014). Measuring the information society report.

[14] Zurovac D, Talisuna AO, Snow RW (2012). Mobile phone text messaging: tool for malaria control in Africa. PLoS Med.

[15] Pop-Eleches C, Thirumurthy H, Habyarimana JP, Zivin JG, Goldstein MP, de Walque D (2011). Mobile phone technologies improve adherence to antiretroviral treatment in a resource-limited setting: a randomized controlled trial of text message reminders. AIDS (London, England).

[16] Lester RT, Ritvo P, Mills EJ, Kariri A, Karanja S, Chung MH (2010). Effects of a mobile phone short message service on antiretroviral treatment adherence in Kenya (WelTel Kenya1): a randomised trial. Lancet.

[17] Mbuagbaw L, Thabane L, Ongolo-Zogo P, Lester RT, Mills EJ, Smieja M (2012). The Cameroon Mobile Phone SMS (CAMPS) trial: a randomized trial of text messaging versus usual care for adherence to antiretroviral therapy. PLoS One.

[18] Mukund Bahadur KC, Murray PJ (2010). Cell phone short messaging service (SMS) for HIV/AIDS in South Africa: a literature review. Stud Health Technol Inform.

[19] Raifman JR, Lanthorn HE, Rokicki S, Fink G (2014). The impact of text message reminders on adherence to antimalarial treatment in northern Ghana: a randomized trial. PLoS One.

[20] Bruxvoort K, Festo C, Kalolella A, Cairns M, Lyaruu P, Kenani M (2014). Cluster randomized trial of text message reminders to retail staff in tanzanian drug shops dispensing artemether-lumefantrine: effect on dispenser knowledge and patient adherence. Am J Trop Med Hyg.

[21] Talisuna AO, Zurovac D, Githinji S, Oburu A, Malinga J, Nyandigisi A (2015). Efficacy of mobile phone short message service (SMS) reminders on malaria treatment adherence and Day 3 post-treatment reviews (SMS-RES-MAL) in Kenya: a study protocol. J Clin Trials.

[22] Onyango EO, Ayodo G, Watsierah CA, Were T, Okumu W, Anyona SB (2012). Factors associated with non-adherence to Artemisinin-based combination therapy (ACT) to malaria in a rural population from holoendemic region of western Kenya. BMC Infect Dis.

[23] Furberg RD, Uhrig JD, Bann CM, Lewis MA, Harris JL, Williams P (2012). Technical implementation of a multi-component, text message-based intervention for persons living with HIV. JMIR Res Protoc.

[24] Republic of Kenya, Siaya County Integrated Development Plan 2013–2017

[25] Noor AM, Kinyoki DK, Ochieng JO, Kabaria CW, Alegana VA, Otieno VA et al (2012) The epidemiology and control profile of malaria in Kenya: reviewing the evidence to guide the future vector control. Division of Malaria Control, Ministry of Public Health and Sanitation & Malaria Public Health Department, KEMRI-Welcome Trust-University of Oxford Research Programme, Oxford

[26] Otieno G, Githinji S, Jones C, Snow RW, Talisuna A, Zurovac D (2014). The feasibility, patterns of use and acceptability of using mobile phone text-messaging to improve treatment adherence and post-treatment review of children with uncomplicated malaria in western Kenya. Malar J.

[27] Medic Mobile. http://www.medicmobile.org

[28] Thirumurthy H, Lester RT (2012). M-health for health behaviour change in resource-limited settings: applications to HIV care and beyond. Bull World Health Organ.

[29] Nhavoto JA, Gronlund A (2015). SMSaude: design, development, and implementation of a remote/mobile patient management system to improve retention in care for HIV/AIDS and tuberculosis patients. JMIR Mhealth Uhealth..

[30] Tomlinson M, Rotheram-Borus MJ, Swartz L, Tsai AC (2013). Scaling up mHealth: where is the evidence?. PLoS Med.

[31] Park LG, Howie-Esquivel J, Dracup K (2014). A quantitative systematic review of the efficacy of mobile phone interventions to improve medication adherence. J Adv Nurs.

[32] Chaquilla WP, Agyepong IA, Ansah E, Gyapong M, Adjei S, Barnish G (2002). Strategies to improve adherence to recommended chloroquine treatment regimes: a quasi-experiment in the context of integrated primary health care delivery in Ghana. JMIR Mhealth Uhealth..

[33] Conteh L, Stevens W, Wiseman V (2007). The role of communication between clients and health care providers: implications for adherence to malaria treatment in rural Gambia. Trop Med Int Health.

[34] Car J, Gurol-Urganci I, de Jongh T, Vodopivec-Jamsek V, Atun R (2012). Mobile phone messaging reminders for attendance at healthcare appointments. Cochrane Database Syst Rev.

[35] Fjeldsoe BS, Marshall AL, Miller YD (2009). Behavior change interventions delivered by mobile telephone short-message service. Am J Prev Med.

[36] Gallagher KM, Updegraff JA (2012). Health message framing effects on attitudes, intentions, and behavior: a meta-analytic review. Ann Behav Med.

[37] Cole-Lewis H, Kershaw T (2010). Text messaging as a tool for behavior change in disease prevention and management. Epidemiol Rev.

